# AF17 Facilitates Dot1a Nuclear Export and Upregulates ENaC-Mediated Na+ Transport in Renal Collecting Duct Cells

**DOI:** 10.1371/journal.pone.0027429

**Published:** 2011-11-08

**Authors:** Hongyu Wu, Lihe Chen, Qiaoling Zhou, Wenzheng Zhang

**Affiliations:** 1 Department of Internal Medicine, The University of Texas Health Science Center at Houston, Houston, Texas, United States of America; 2 Graduate School of Biomedical Sciences, The University of Texas Health Science Center at Houston, Houston, Texas, United States of America; 3 Department of Internal Medicine, Xiangya Hospital, Central South University, Changsha, Hunan, People's Republic of China; INSERM, France

## Abstract

Our previous work in 293T cells and *AF17^-/-^* mice suggests that AF17 upregulates expression and activity of the epithelial Na^+^ channel (ENaC), possibly by relieving Dot1a-AF9-mediated repression. However, whether and how AF17 directly regulates Dot1a cellular distribution and ENaC function in renal collecting duct cells remain unaddressed. Here, we report our findings in mouse cortical collecting duct M-1 cells that overexpression of AF17 led to preferential distribution of Dot1a in the cytoplasm. This effect could be blocked by nuclear export inhibitor leptomycin B. siRNA-mediated depletion of AF17 caused nuclear accumulation of Dot1a. AF17 overexpression elicited multiple effects that are reminiscent of aldosterone action. These effects include 1) increased mRNA and protein expression of the three *ENaC* subunits (α, β and γ) and serum- and glucocorticoid inducible kinase 1, as revealed by real-time RT-qPCR and immunoblotting analyses; 2) impaired Dot1a-AF9 interaction and H3 K79 methylation at the *αENaC* promoter without affecting AF9 binding to the promoter, as evidenced by chromatin immunoprecipitation; and 3) elevated ENaC-mediated Na^+^ transport, as analyzed by measurement of benzamil-sensitive intracellular [Na^+^] and equivalent short circuit current using single-cell fluorescence imaging and an epithelial Volt-ohmmeter, respectively. Knockdown of AF17 elicited opposite effects. However, combination of AF17 overexpression or depletion with aldosterone treatment did not cause an additive effect on mRNA expression of the ENaC subunits. Taken together, we conclude that AF17 promotes Dot1a nuclear export and upregulates basal, but not aldosterone-stimulated ENaC expression, leading to an increase in ENaC-mediated Na^+^ transport in renal collecting duct cells.

## Introduction

The primary function of the renal collecting duct is to adjust the final urinary solute osmolarity and concentrations, and is comprised of two functionally distinct epithelial cells: the principal and intercalated cells [1]. The principal cells of the cortical collecting duct contribute significantly to this process [2], [3]. Na^+^ is reabsorbed from the renal ultrafiltrate through the epithelial Na^+^ channel (ENaC) and the Na^+^/Cl^–^ cotransporter at the apical side, and excreted at the basolateral membrane of the principal cells into the blood by the Na^+^/K^+^ ATPase. Na^+^ absorption concurrent with the osmotic movement of water increases extracellular fluid volume and consequently blood pressure [4]. Many groups have studied the molecular mechanisms governing ENaC trafficking, cell surface expression, maturation, assembly, open probability, and degradation [5], [6], [7], [8], [9]. For example, studies have shown that functional ENaC is assembled from three structurally related subunits (α, β, γ) in the endoplasmic reticulum, where it is subject to *N*-linked glycosylation [10], [11], [12]. The maturation of the channel requires further posttranslational modifications on its passage through the Golgi. These modifications include furin-mediated cleavage of α and γ subunits and the concurrent substitution of a high-mannose glycosylation pattern for a complex one [13]. The glycosylation and proteolytic processing of the ENaC channel are prerequisites for full activity.

However, the mechanisms controlling ENaC transcription, especially in the context of chromatin, are not well defined. Aldosterone imposes tight and complex regulation on ENaC at both transcriptional and posttranscriptional levels. The classical mechanism of aldosterone action involves binding to the cytoplasmic mineralocorticoid receptor (MR), which functions as a ligand-dependent transcription factor. Upon binding of the ligand, MR is translocated into the nucleus and stimulates transcription by binding to the hormone response elements present in the 5′ flanking regions of target genes [14]. Nevertheless, how the ligand-bound MR gains access to DNA that is packed into chromatin remains largely unknown.

Using mouse inner medullary collecting duct mIMCD-3 cells and kidneys isolated from Sgk1 WT and mutant mice, we have previously found that disruptor of telomeric silencing alternative splice variant a (Dot1a) [15] and ALL-1 fused gene from chromosome 9 (AF9) [16] form a protein complex that represses *αENaC* in an aldosterone-sensitive manner. Under basal conditions, Dot1a-AF9 binds to the specific subregions of *αENaC* promoter, promotes H3 K79 methylation, and inhibits transcription [15], [16]. Aldosterone relieves the repression by decreasing mRNA expression of Dot1a and AF9, and by impairing Dot1a-AF9 interaction through Sgk1-mediated AF9 phosphorylation at Ser435 [17]. Hence, transcriptional activation of α*ENaC* by aldosterone can be partially attributed to induction of Sgk1 and downregulation of Dot1a and AF9 mRNA expression.

Recently, we found that ALL-1 partner at 17q21 (AF17) functions as a competitor of AF9 to bind the same domain of Dot1a and enhances ENaC-mediated Na^+^ transport in 293 cells [18]. We also generated the first (to our knowledge) *AF17^-/-^* mice and identified their phenotype characterized by increased urinary Na^+^ excretion and decreased blood pressure [19]. While all of these studies suggest the importance of AF17 in relieving Dot1a-AF9-mediated repression of ENaC genes, and tuning ENaC-mediated Na^+^ transport and blood pressure, evidence demonstrating that AF17 plays these roles in the renal collecting duct cells, the physiological site of ENaC-mediated Na^+^ transport in the kidney, is still missing. In addition, the effect of AF17 overexpression on ENaC induction by aldosterone remains unknown.

In this report, we primarily use mouse cortical collecting duct M-1 cells as the model system to address these questions. We found that AF17 promotes Dot1a cytoplasmic expression in a leptomycin B (a nuclear export inhibitor)-sensitive manner, and increases ENaC expression and activity in these more physiologically relevant cells.

## Results

### AF17 enhances Dot1a nuclear export in a leptomycin B-sensitive manner in M-1 cells

**Figure 1:**
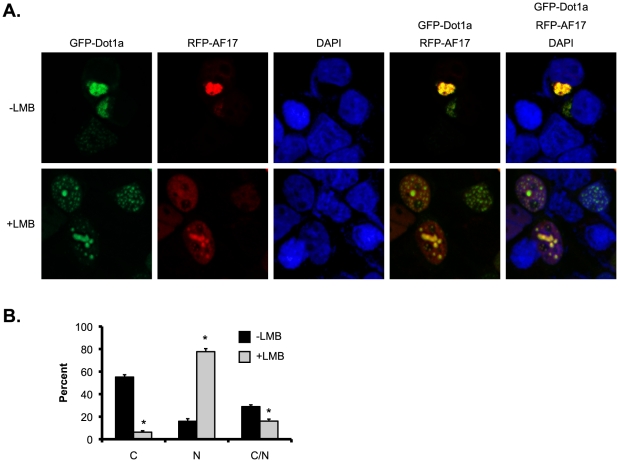
Inhibition of nuclear export by LMB promotes nuclear accumulation and cytoplasmic depletion of Dot1a-AF17 complex in M-1 cells. *A*. Representative deconvolution microscopy images show cytoplasmic or nuclear colocalization of transiently expressed GFP-Dot1a and RFP-hAF17 in the absence (top panel) or presence (low panel) of LMB (10 nM) in M-1 cells. Original amplification: X400. Note: Dot1a in the low panel exhibited the typical nuclear distribution pattern characterized by large discrete foci. *B*. The bar graph shows that LMB causes preferential expression of Dot1a and AF17 in the nucleus. As in *A* except for that cells expressing both of GFP-Dot1a and RFP-AF17 were examined by epifluorescence microscopy and categorized as cytoplasmic (*C*), nuclear (*N*), or both (C/N) depending on the location of the fusion proteins. The graphed value (%) is the number of cells of each localization type divided by the total number of cells examined. At least 250 cotransfected cells were examined from three independent experiments (*n* = 3). Each percentage was compared with control (-LMB) within the category. *n* = 3. *: *p*<0.05.

To demonstrate the biological relevance of the Dot1a-AF17 interaction and to directly test the theory that AF17 facilitates Dot1a nuclear export, we chose M-1 cells as the model system. M-1 cells were derived from mouse cortical collecting duct, and are frequently used for investigation of ENaC mRNA and protein expression as well as on ENaC-mediated Na+ transport by many groups and us [20], [21], [22], [23]. Accordingly, M-1 cells were cotransfected with two constructs expressing red fluorescence protein-tagged (RFP)-hAF17 and GFP-Dot1a, treated with methanol as vehicle control or leptomycin B (LMB, 10 nM) to specifically inhibit CRM-1-mediated nuclear export. This concentration of LMB was selected because it has been shown to inhibit nucleocytoplasmic shuttling of Id1 in human umbilical vein endothelial cells [24]. The cellular distribution of GFP-Dot1a and RFP-hAF17 was then determined by deconvolution microscopy. In the absence of LMB, GFP-Dot1a and RFP-hAF17 were primarily, if not exclusively, located in the cytoplasm and colocalized (Fig. 1A, top panel). The typical nuclear distribution pattern of GFP-Dot1a when expressed alone or in combination with RFP-AF9 is large discrete foci throughout the nucleus [16]. This pattern was hardly found in these methanol-treated cells. However, addition of LMB promoted the typical nuclear distribution of Dot1a. Furthermore, the majority of RFP-hAF17 also resided in the nuclei and colocalized with GFP-Dot1a (Fig 1A, bottom panel).

To more accurately assess the effects of AF17 overexpression and LMB on Dot1a cellular distribution, the cotransfected cells were divided into three categories based on the cellular distribution of RFP-hAF17 and GFP-Dot1a. Unlike the two types mentioned above, the third type of expression pattern was seen in cells that displayed substantial signals in both the cytoplasm and the nucleus. Without LMB, the two fusion proteins resided in the cytoplasm in about 55% of cells. Their nuclear expression was observed in only 16% of cells. The remaining 29% of cells displayed substantial GFP-Dot1a and RFP-hAF17 in both of the cytoplasm and nucleus (Fig. 1B). In the presence of LMB, the percentage of the cytoplasmic pattern was reduced from 55% to only 6% of cells. In contrast, the percentage of the cells displaying Dot1a and AF17 nuclear expression was increased from 16% to 77% (Fig. 1B). These observations suggest that overexpression of RFP-hAF17 and GFP-Dot1a leads to preferential shift of both proteins from the nucleus to the cytoplasm, most likely through mechanisms involving CRM-1-mediated nuclear export that can be blocked by LMB.

Since GFP or RFP may alter the subcellular localization of tagged proteins, we examined the effect of WT Dot1a on RFP-hAF17 localization and reciprocally the effect of hAF17 on GFP-Dot1a localization. Overexpression of WT Dot1a had marginal effect on RFP-hAF17 cellular distribution as evidenced by no significant differences in each category between Vec- and Dot1a-transfected cells (Fig. S1A). In contrast, the cellular distribution of GFP-Dot1a was significantly impacted by coexpression of WT hAF17, with cytoplasmic expression being increased from ∼10% to ∼50% and nuclear expression reduced from ∼70% to ∼22% (Fig. S1B).

**Figure 2:**
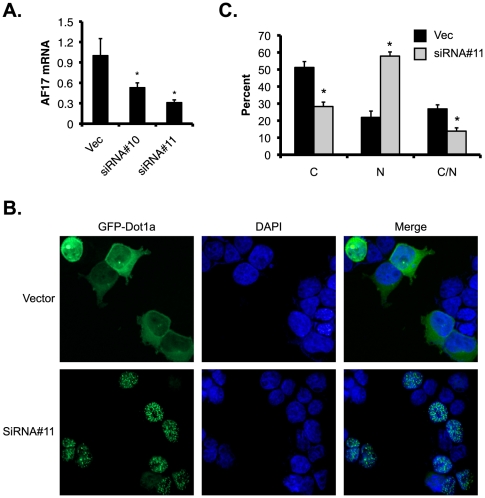
Depletion of AF17 enhances nuclear distribution of Dot1a in M-1 cells. ***A.*** M-1 cells were stably transfected with pSilencer-2.1-U6-Hygro vector (Vec) or its derivatives bearing AF17-specific siRNA#10 or siRNA#11. Total RNA was analyzed by real-time RT-qPCR for AF17 expression. ***B-C.*** M-1 cells stably transfected with the vector or siRNA#11 as shown in ***A*** were transiently transfected with pGFP-Dot1a and analyzed by deconvolution microscopy as in Fig. 1A (***B***) or epifluorescence microscopy as in Fig. 1B (***C***). Original amplification: X400. *n* = 3. *: *p*<0.05 vs. vector in each category.

In reciprocal experiments, we applied RNA interference technology to deplete the endogenous AF17 and examined the effects on the cellular distribution of GFP-Dot1a. Two siRNA constructs specifically targeting AF17 and a negative control construct were transfected into M-1 cells to establish stable cell lines. No adverse effects on cell growth or morphology were observed in any of the siRNA-transfected cell lines. Real-time RT-qPCR showed that siRNA#10 and siRNA#11 knocked down AF17 mRNA levels to 53% and 31%, respectively, compared with the control cell line transfected with the negative control construct harboring an unrelated siRNA target sequence (Fig. 2A). Since siRNA#11-transfected cells had more efficiently depleted AF17 expression, these cells along with the control cell line were transiently transfected with the GFP-Dot1a construct. In the control cell line, GFP-Dot1a displayed the cytoplasmic expression pattern in 51% of cells, with 22% of cells expressing GFP-Dot1a in the nucleus or 27% of cells in both of the compartments. In the siRNA#11-transfected cells, these numbers were significantly changed into 28%, 58% and 14%, respectively (Fig. 2B and 2C). In brief, our data are consistent with the notion that AF17 promotes distribution of Dot1a from the nucleus to the cytoplasm, most likely through CRM-1-mediated nuclear export pathway.

### AF17 overexpression impairs H3 K79 methylation at the *αENaC* promoter in M-1 cells

**Figure 3:**
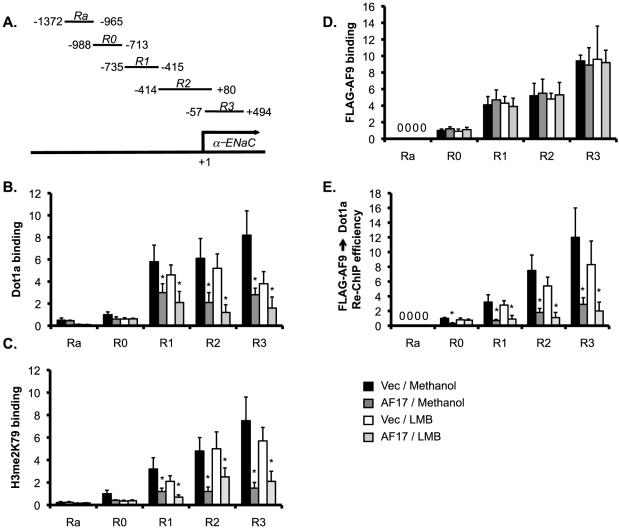
AF17 impairs Dot1a-AF9 interaction and H3 K79 methylation at the *αENaC* promoter in M-1 cells. A. Diagram of the *αENaC* promoter [17]. ***B-E***. Chromatin immunoprecipitation (ChIP) and sequential ChIP (Re-ChIP) assays demonstrating that overexpression of AF17 differentially affected the abundance of Dot1a (***B***), H3 dimethylated K79 (H3 me2K79) (***C***), FLAG-AF9 (***D***), and FLAG-AF9 interaction with Dot1a (***E***) at the *αENaC* promoter. M-1 cells were transiently transfected with pFLAG-AF9 along with pcDNA3.1 (Vec) or pcDNA-AF17 (AF17). 6 h later, the cells were treated with vehicle or LMB (10 nM) for an additional 16 h. Chromatin was immunoprecipitated by the antibodies as indicated, followed by real-time qPCR with primers amplifying Ra and R0-R3 subregions of the *αENaC* promoter as shown in ***A***. For Re-ChIP, chromatin was sequentially immunoprecipitated with anti-FLAG and anti-Dot1a antibodies. Relative ChIP or Re-ChIP efficiency was defined as the (re-) immunoprecipitated amount of materials present as compared to that of the initial input sample, and set to 1 in R0 from the Vec-transfected cells treated with vehicle, and was calculated accordingly for all other samples. *: P<0.05 vs. Vec within the same subregion for the same treatment. n = 3–4 for all panels.

We previously demonstrated that the Dot1a-AF9 complex is associated with specific subregions of the *αENaC* promoter and promotes H3 K79 hypermethylation at these subregions in mIMCD-3 cells [15], [16], [17]. Given the facts that AF17 facilitates Dot1a nuclear export (Fig. 1 and 2), we intended to determine if AF17-mediated downregulation of Dot1a nuclear expression is coupled to changes in Dot1a-AF9 interaction and H3 K79 methylation associated with the *αENaC* promoter. M-1 cells were transiently transfected with pFLAG-AF9 (to determine AF9 binding and its interaction with Dot1a at the promoter) along with pCDNA3.1 vector as control or pCDNA-AF17, followed by incubation with LMB or methanol as vehicle control. The resulting four groups of cells were then analyzed by chromatin immunoprecipitation coupled real-time qPCR (ChIP-qPCR) with specific primers for amplification of the five subregions of the *αENaC* promoter (Fig. 3A).

ChIP with antibodies against Dot1a or H3 me2K79 revealed relatively higher levels of Dot1a, and thus elevated H3 me2K79 associated with R1-R3, as compared to Ra and R0 subregions in all groups (Fig. 3B and 3C), similar to what we reported in mIMCD-3 cells [17]. AF17 overexpression significantly decreased the association of Dot1a and thus H3 me2K79 with R1-R3 to various degrees, compared to those in the vector-transfected cells (Fig. 3B and 3C) in the absence or presence of LMB. These data suggest that AF17 regulates Dot1a and H3 me2K79 at the *αENaC* promoter in M-1 cells. Taken together with the subcellular localization data (Fig. 1 and 2), we speculate two mechanisms. Without inhibition of nuclear export by LMB, overexpressed AF17 may promote Dot1a nuclear export, leading to impairment of nuclear-located and thus promoter-associated H3 K79 methylation. Addition of LMB may cause the majority of overexpressed AF17 and Dot1a located in the nucleus where AF17 inhibits Dot1a-AF9 interaction at the promoter (Fig. 3 D and E, see below).

To directly test the hypothesis that AF17 overexpression inhibits Dot1a-AF9 interaction at the *αENaC* promoter, ChIP with anti-FLAG was performed. Interaction of AF9 with R0-R3, but not with Ra, was detected. Within each of the subregions, the four groups of cells were indistinguishable in terms of AF9 binding (Fig. 3D). However, sequential ChIP first with the anti-FLAG antibody coupled with an anti-Dot1a antibody (Re-ChIP) revealed that AF17 overexpression significantly impaired the Dot1a-AF9 interaction at all of the four subregions (R0-R3), regardless of the LMB treatment (Fig. 3E). Therefore, like aldosterone-induced Sgk1 [17], AF17 appears to regulate the Dot1a-AF9 interaction at the promoter without measurably affecting the association of AF9 with the promoter.

### AF17 expression is not regulated by aldosterone in M-1 and mIMCD-3 cells

We have reported that aldosterone downregulates mRNA expression of Dot1a and AF9 in mIMCD-3 cells. However, the effect of aldosterone on AF17 mRNA expression in M-1 as well as in mIMCD-3 cells remains unknown. Accordingly, real-time RT-qPCR of M-1 cells treated with aldosterone (1 µM for 24 h) or vehicle was conducted. ENaC, Dot1a and AF9 genes were included as controls of aldosterone-upregulated and -downregulated genes, respectively. As expected, aldosterone significantly stimulated mRNA expression of the three ENaC genes, with their mRNA levels being elevated to 410%, 256%, and 187% of control, respectively (Fig. S2A). Dot1a and AF9 mRNA levels were significantly lowered to 19% and 25% of control by aldosterone treatment (Fig. S2B). However, no significant alteration in AF17 mRNA abundance was seen (Fig. S2B).

In parallel experiments, mIMCD-3 cells were treated with aldosterone (1 µM for 24 h) or vehicle, and analyzed by RT-qPCR. While aldosterone significantly increased ENaC expression at both mRNA and protein levels [23], and decreased Dot1a and AF9 mRNA levels ([15], [16] and Fig. S2C), it failed to substantially affect AF17 mRNA expression (Fig. S2C). We conclude that AF17 is most likely not regulated by aldosterone at the transcriptional level in M-1 and mIMCD-3 cells under the conditions tested.

### AF17 plays a redundant role with aldosterone in upregulating *αENaC* expression in M-1 cells

**Figure 4:**
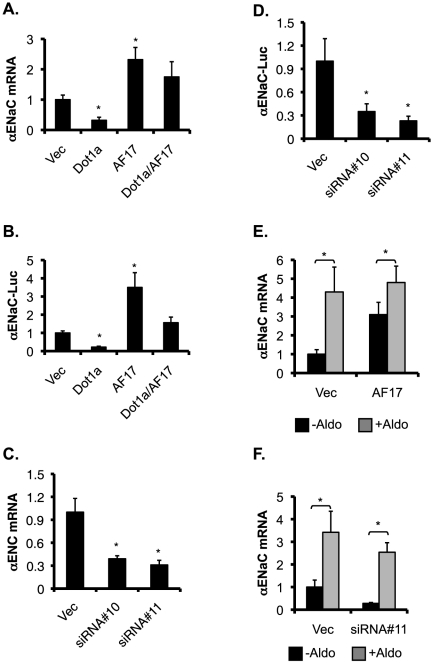
AF17 upregulates basal, but not aldosterone-stimulated expression of *αENaC* in M-1 cells. ***A-B***. AF17 overexpression increases basal *αENaC* transcription. M-1 cells were transiently transfected with pGL3Zeocin-1.3-*αENaC* along with pcDNA3.1 (Vec) or its derivatives expressing Dot1a or AF17. The total amount of plasmid DNA was kept constant for all transfections. Total RNA was analyzed for *αENaC* and actin expression by real-time RT-qPCR. *P<0.05. n = 3 (***A***). Alternatively, whole cell lysates were prepared and luciferase reporter assays were performed. *P<0.05 vs. Vec. n = 4 (***B***). ***C-D.*** Knockdown of AF17 mRNA expression decreases basal *αENaC* transcription. M-1 cells were stably transfected with pSilencer-2.1-U6-Hygro vector (Vec) or its derivatives bearing AF17-specific siRNA#10 or siRNA#11. Total RNA was analyzed by real-time RT-qPCR for AF17 (see Fig. 2A) or *αENaC* (***C***) as in ***A***, and whole cell lysates were examined by luciferase assays (***D***) as in ***B***. In all cases, n = 3. *: P<0.05 vs. vector. ***E-F***. AF17 overexpression or knockdown had marginal effects on the aldosterone-mediated induction of *αENaC* expression. Stably transfected M-1 cells overexpressing AF17 (see Fig. 8A) or depleting AF17 (see Fig. 2A) were treated with ethanol as vehicle control (-Aldo) or aldosterone (+Aldo, 1 µM), and analyzed by RT-qPCR for αENaC as in ***A***. In all cases, n = 3. *: P<0.05 vs. vector.

Since AF17 impairs Dot1a-AF9 interaction and H3 K79 methylation at the *αENaC* promoter, we anticipate that AF17, like aldosterone, relieves Dot1a-mediated repression of the *αENaC* promoter in M-1 cells. To test this hypothesis, M-1 cells were transiently transfected with pcDNA3.1, pcDNA-Dot1a, or pcDNA-AF17 along with an *αENaC* promoter luciferase construct, and examined by real-time RT-qPCR and luciferase assay. β-actin was used as an internal control in RT-qPCR. *αENaC* mRNA was ∼32%, 230%, or 175% of the control in cells overexpressing Dot1a, AF17, or both, respectively (Fig. 4A). A similar pattern was obtained for the expression of the luciferase reporter (Fig. 4B).

In reciprocal experiments, we examined the effects of AF17 depletion on the activity of the *αENaC* promoter. AF17 knockdown in siRNA#10- and siRNA#11-transfected M-1 cells was accompanied with a reduction of *αENaC* mRNA levels to 39% and 31% of control, respectively (Fig. 2A and 4C). Similarly, the luciferase reporter activity was also significantly lowered in these cells, compared to control (Fig. 4D). Consistently, *αENaC* expression at the protein level was also upregulated by AF17 overexpression and downregulated by AF17 knockdown (see below).

To investigate the effect of AF17 overexpression on the aldosterone-induction of *αENaC* mRNA expression, M-1 cells were stably transfected with pCDNA3.1 (Vec) or pCDNA-hAF17 (AF17). These stable cells were treated with aldosterone (1 µM for 24 h) or vehicle and analyzed by RT-qPCR. The *αENaC* mRNA levels was significantly elevated to 430% and 310% of the control (vehicle-treated, Vec-transfected cells) by aldosterone and AF17 overexpression, respectively (Fig. 4D). However, the effect of AF17 overexpression on the aldosterone-induction of *αENaC* mRNA expression was marginal, because *αENaC* mRNA level was only further increased to 480% of control) in the AF17-overexpressing, aldosterone-treated cells (Fig. 4D). Knockdown of AF17 also had subtle effect on *αENaC* induction by aldosterone (Fig. 4E). Similar results were obtained when β and γENaC mRNA levels were examined in parallel (Fig. S3A and S3B). In brief, aldosterone and AF17 may play a redundant role in regulating mRNA expression of the ENaC subunits.

### AF17 regulates several other aldosterone target genes in M-1 cells

**Figure 5:**
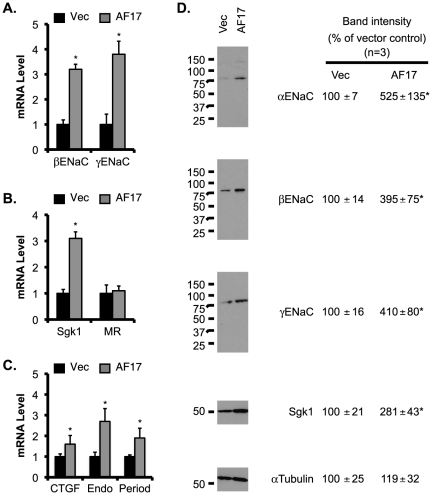
Overexpression of AF17 increases mRNA and protein expression of ENaC and Sgk1 in M-1 cells. M-1 cells were transiently transfected with pcDNA3.1 (Vec) or pcDNA-AF17 (AF17), and analyzed by RT-qPCR as in Fig. 4A. Shown are *βENaC* and *γENaC* (***A***), ENaC regulators *Sgk1* and *MR* (***B***), three other aldosterone target genes: *CTGF*, *preproendothelin-1*, and *period* (***C***). In ***D***, whole cell lysate was analyzed by immunoblotting with antibodies against the proteins indicated. The relative abundance of mRNA or protein of each gene was set to 1 or 100, respectively, in vector-transfected cells, and used for comparison. n = 3. *: P<0.05 vs. Vector (Vec) for each gene.

To determine if AF17 plays a role in the transcriptional control of other aldosterone target genes in M-1 cells, we used RT-qPCR to analyze *βENaC*, *γENaC*, *Sgk1*, *MR*, *CTGF*, *preproendoethelin-1*, and *period*, all except MR had significantly increased mRNA expression due to AF17 overexpression to various degrees (Fig. 5A-C).

To determine whether AF17-mediated transcriptional alteration of these target genes is translated into corresponding changes in their protein abundance, immunoblotting analyses were performed. As shown in Fig. 5D, the protein abundance of αENaC, βENaC, γENaC, and Sgk1 were 2–4 fold higher in the AF17-overexpressing cells than in the control.

**Figure 6:**
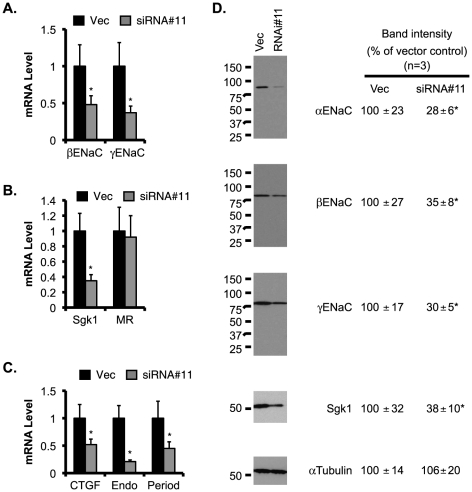
Knockdown of AF17 decreases mRNA and protein expression of ENaC and Sgk1 in M-1 cells. M-1 cells were stably transfected with pSilencer-2.1-U6-Hygro vector (Vec) or its derivative bearing AF17-specific siRNA#11, and analyzed by RT-qPCR (***A*** *-* ***C***) or immunoblotting (***D***) as in Fig. 5. n = 3. *: P<0.05 vs. Vector (Vec) for each gene.

Since AF17 mRNA was more effectively depleted in siRNA#11-transfected cells, we used these cells to determine if AF17 knockdown yields an opposite effect on expression of these aldosterone target genes. As expected, a significant reduction in the mRNA levels of βENaC, γENaC, Sgk1, CTGF, preproendoethelin-1, and period was found in siRNA#11-transfected cells, as compared to the control (Fig. 6A-C). AF17 depletion had little effect on MR mRNA expression. Furthermore, the impaired mRNA expression was followed by a decrease of the protein abundance of each corresponding gene examined (Fig 6D).

### AF17 enhances benzamil-sensitive Na^+^ transport in M-1 cells

**Figure 7:**
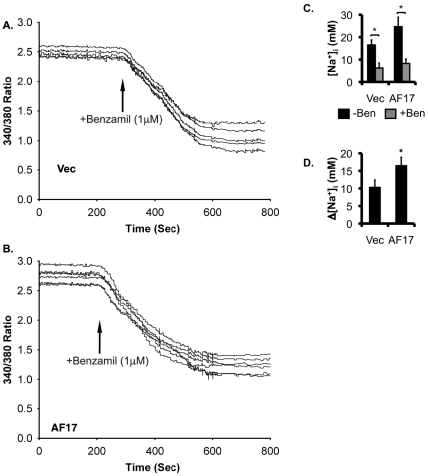
Overexpression oAF17 enhances extracellular [Na^+^]_i_ in M-1 cells. ***A-B.*** Shown are representative SBFI recordings of M-1 cells transiently transfected with red fluorescence protein vector RFP (Vec) or RFP-AF17. The cells were analyzed for Na^+^ transport. Transfected cells were first identified and marked by epifluorescence microscopy with an RFP-specific filter. The same field of cells was then switched to SBFI-specific filters for [Na^+^]_i_ imaging. ***C-D.*** Shown are the averages of [Na^+^]_i_ before (-Ben) and after (+Ben) 1 µM benzamil addition (***C***) and benzamil-sensitive [Na^+^]_i_ (***D***) from at least 25 transfected cells per transfection from three independent experiments. Readings of non-transfected cells were excluded from analysis. In all cases, *: P<0.05 vs. Vec. n = 3.

To determine if AF17-mediated regulation of mRNA and protein expression of ENaC genes is physiologically coupled to ENaC activity, we performed SBFI-AM-based single cell fluorescence imaging to measure intracellular [Na^+^] ([Na^+^]_i_). M-1 cells were transiently transfected with RFP as vector control or RFP-hAF17. Transfected cells were identified by epifluorescence microscopy and subjected to measurement of [Na^+^]_i_. Representative tracings of the two transfections are given in Fig. 7A-B, respectively. The basal level of [Na^+^]_i_ (in mM, the same below) was decreased from 16.5 to 6.23 by addition of benzamil in vector-transfected cells, suggesting that ENaC is primarily responsible for Na^+^ transport in this cell line. These figures became 24.7 and 8.2, respectively, in AF17-overexpressing cells (Fig. 7C). Therefore, the corresponding benzamil-sensitive [Na^+^]_i_ was significantly elevated from 10.3 to 16.5 by AF17 overexpression (Fig. 7D).

**Figure 8:**
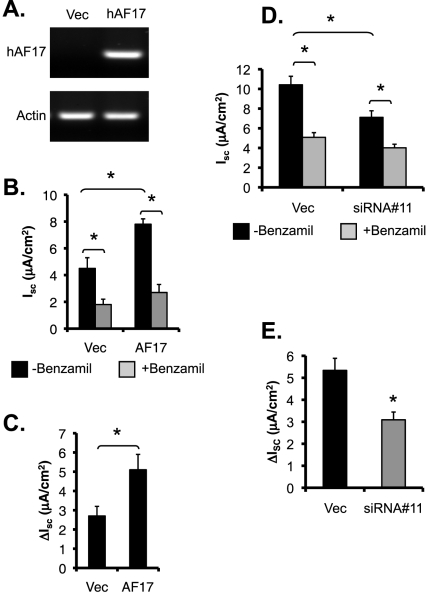
AF17 enhances benzamil-sensitive equivalent short-circuit current (*I*_sc_) in M-1 cells. ***A*** **.** Representative agarose gel analyses of hAF17 expression in M-1 cells stably transfected with pCDNA3.1 (Vec) or pcDNA-hAF17. Cells were grown on permeable filters and allowed to form confluent monolayers. Total RNA was prepared and examined by RT-PCR with primers specific for hAF17, followed by agarose gel analysis. ***B-C.*** As in *A*, shown are averages of *I*_sc_ before (-Benzamil) and after (+Benzamil) addition of 1 µM benzamil (*B*), or benzamil-sensitive *I*_sc_ (ΔI_sc_; *C*). *P<0.05 vs. Vec. *n* = 12 or 15 for each cell population, respectively. ***D-E.*** Results are as in ***B-C*** except for that M-1 cells stably depleting AF17 as shown in Fig. 2A were used. *P<0.05 vs. Vec. n = 14 and 17, respectively.

Measurement of the equivalent short circuit current (I_sc_) was carried out to independently confirm these results. M-1 cells were stably transfected with pCDNA3.1 or pCDNA-hAF17. To determine whether the cells cultured on permeable supports express hAF17, primers were designed to specifically amplify hAF17, but not the endogenous mouse Af17. hAF17 was readily detectable in the pCDNA3.1-hAF17 transfected cells, and undetectable in the pCDNA3.1-transfected cells, as evidenced by DNA agarose gel analysis of RT-PCR with these hAF17-specific primers (Fig. 8A). Similar to previous findings (Helms et al, 2003), these two cell lines developed comparably high transepithelial resistance (1001 vs. 1150 Ω.cm^2^) (Fig. S4). In the absence of benzamil, I_sc_ (in µA/cm^2^) was 4.5 or 7.8 in the vector-transfected or AF17-overexpressing cells, respectively. Addition of benzamil dramatically inhibited I_sc_ to 1.8 and 2.7 (Fig. 8B), suggesting that ENaC is the primary, if not exclusive, target in AF17-mediated regulation of Na^+^ transport in M-1 cells. It should be noted that our measurements of transepithelial resistance and I_sc_ are comparable to those measured for M-1 cells by others (Helms et al, 2003). Collectively, the data consistently indicate that AF17 impairs Dot1a-AF9 mediated suppression of ENaC transcription, leading to enhanced ENaC mRNA and protein expression and subsequent ENaC activity.

## Discussion

Our earlier work, primarily done in mIMCD-3 cells, led to identification and characterization of an aldosterone-signaling network controlling *αENaC* transcription [15], [16], [17]. The network involves Dot1a, AF9, and Sgk1. Recently, we added AF17 into this network, characterized its role first in vitro in 293T cells [18] and then in vivo in mouse kidney [19]. While 293T cells provide practical advantages in transient transfection studies of heterologous genes in a kidney epithelial cell type, they are not specifically generated from the renal collecting duct, where ENaC-mediated physiological Na^+^ transport in the kidney occurs. Analyses of kidneys from both WT and AF17^-/-^ mice provided strong evidence of AF17 involvement in Na^+^ metabolism and blood pressure control. Nevertheless, the complexity of the whole animal context and heterogeneous cellular composition of the kidney makes it impractical to obtain direct evidence showing the function and regulation of AF17 in renal collecting duct. Therefore, in this report, we pursue to define AF17 role in a more homogenous, physiologically relevant system: M-1 cells.

The M-1 cells were derived from renal cortical collecting duct (CCD) micro-dissected from a transgenic mouse carrying the early region of SV40 virus [25]. The CCD is characterized by expression of CCD-specific antigens, a high transepithelial electrical resistance, a luminal-negative transepithelial potential difference, ion transport primarily through ENaC-mediated Na+ transport that can be blocked by amiloride, and responsiveness to aldosterone and arginine vasopressin [25]. These features are apparently maintained in M-1 cells [25]. With multiple approaches, we solidify the notion of AF17 as a regulator of Dot1a nuclear/cytoplasmic distribution, ENaC expression, and ENaC-mediated Na^+^ absorption in mammalian collecting duct cells. In particular, we provide the first line of evidence suggesting that M-1 cells may share the same mechanisms with mIMCD-3 cells to control *αENaC* transcription through Dot1-AF9-AF17-mediated H3 K79 methylation.

AF17 and Sgk1 share some functional aspects. In particular, they regulate *αENaC* transcription at least in part by modulating H3 K79 methylation at the *αENaC* promoter. ChIP and Re-ChIP demonstrate that in the absence of LMB, the association of Dot1a with R1-R3 subregions of *αENaC* promoter was 48% to 65% lower than control in AF17 overexpressing cells. The decreased Dot1a binding was followed by a reduction of 60% to 80% in H3 K79 methylation in these regions. The effects of AF17 overexpression were not generally affected by LMB. In all cases, AF9 binding to the promoter was not significantly impaired. These observations are reminiscent of what we observed in mIMCD-3 cells when Sgk1 was overexpressed [17].

However, AF17 and Sgk1 differ in their mechanisms. First, AF17 impairs Dot1a-AF9 interaction by competing with AF9 for the same binding domain within Dot1a [18]. Sgk1 attenuates Dot1a-AF9 complex formation by decreasing AF9 ability to interact with Dot1a through phosphorylating AF9 Ser435 [17]; Secondly, AF17 association with the *αENaC* promoter has not been established because of the difficulties in detecting AF17 in immuoprecipitation/immunoblotting analyses as we discussed in detail before [18]. Whether AF17 regulates the promoter-bound or promoter-free Dot1-AF9 complex remains to be defined. In contrast, Sgk1 is shown to bind *αENaC* promoter [17]. Its interference with Dot1a-AF9 interaction can presumably occur even at the *αENaC* promoter; Thirdly, AF17 mRNA expression is not likely to be regulated by aldosterone, at least in the three cell lines examined (293T, M-1, and mIMCD-3) under the conditions tested (1 µM for 24 hr), while Sgk1 is rapidly and persistently induced by aldosterone [17], [26]; Fourthly, AF17 regulates Sgk1 mRNA and protein expression (Fig. 5B & D). Given the fact that Sgk1 is involved in many signaling pathways, and its expression can be induced by multiple stimuli [27], it can be postulated that AF17 may also play a role in these signaling pathways and functions upstream of Sgk1. It can be speculated that AF17 relieves Dot1a-AF9 repression not only by competing with AF9 to bind Dot1a, but also through increasing Sgk1 expression to enhance Sgk1-mediated AF9 phosphorylation. Future studies are needed to address the later possibility.

Previous studies from different groups suggest that aldosterone regulate ENaC expression in a subunit- and tissue-specific manner. Classically, aldosterone does not stimulate β and γENaC expression in the renal cortical collecting duct (reviewed in [28]). For example, elevated circulating aldosterone as a result of either dietary NaCl restriction or aldosterone infusion selectively increased αENaC protein abundance without increasing the levels of the β and γ subunits in rat kidney [29]. This pattern was changed in the colon, where β and γ ENaC mRNA levels were elevated by aldosterone administration while αENaC was expressed constitutively [30], [31], [32]. Data from cultured cell lines are also controversial. In mCCD cells, aldosterone (300 nM, 24 hr) significantly increased α and γENaC expression at both mRNA and protein levels, whereas had little impact on βENaC expression [33]. In the wild type or protein kinase D1-depleted M-1 cells, aldosterone (10 nM, 4hr) did not significantly alter βENaC protein expression [20]. In the A6 cells, which are believed to have similar signal transduction and electrophysiological properties to those of mammalian collecting duct principal cells, aldosterone (100 nM, 3h or 6h) stimulated mRNA synthesis of α and γ, but not β subunit. However, at the protein level, only α and β subunits were increased by the aldosterone treatment at these time points [34]. This result differs from those reported by others in the A6 cells. For instance, β but not α and γENaC protein levels were reproducibly increased in response to long-term administration of aldosterone (1.5 µM; ≥72 h) [35]. In another study with the A6 cells, aldosterone (300 nM, 24 h) was shown to increase mRNA abundance of the three ENaC subunits [36]. The induction of the α, β and γENaC by aldosterone (1 µM, 24 h) was also observed in 293T and mIMCD3 cells in our previous studies [18], [23] and in M-1 cells in the current study (Fig. 4 and S3). It can be speculated that multiple factors may contribute to the inconsistent observations described above. These factors include the homogeneity/heterogeneity of the cell population involved, and the doses and time points of aldosterone treatment. It also should be noted that the respective roles of MR and GR in mediating the effects of aldosterone, particularly at high concentrations (such as 1 µM), remain to be defined.

## Materials and Methods

Rabbit polyclonal antibodies specific for ENaC subunits α, β, γ, Dot1a, FLAG, and Sgk1 as well as the plasmids expressing untagged Dot1a and AF17 (pcDNA-Dot1a and pcDNA-hAF17), green or red fluorescence-tagged Dot1a (GFP-Dot1a and RFP-hAF17), AF17-specific siRNA#10 or siRNA#11 have been described in our previous reports [15], [16], [17], [18], [23]. All chemicals including benzamil, nigericin, monensin, benzofuran isophthalate acetoxymethyl ester (SBFI-AM) and aldosterone were purchased from Sigma (St. Louis, MO, USA).

### Cell culture, transient and stable transfection and aldosterone treatment

mIMCD-3 and M-1 cells (American Type Culture Collection, Manassas, VA, USA) were grown in DMEM/F12 plus 10% FBS. Cells were seeded in DMEM/F12 plus 10% charcoal-stripped FBS for at least 50 hours, followed by addition of 1 µM aldosterone or 0.01% ethanol as vehicle control for 24 h. LIPOFECTAMINE^TM^ 2000 reagent (Invitrogen) was routinely used for transient transfection. To deplete AF17 mRNA levels by RNA interference, M-1 and mIMCD-3 cells were transfected by siRNA#10, siRNA#11, and the parent vector pSilencer-2.1-U6-Hygro as a negative control, and selected by hygromycin (500 g/ml) treatment for about 2 weeks. All surviving colonies from the same transfection were combined and expanded to establish the corresponding cell lines. Parent cells were treated with hygromycin similarly in parallel to assess the selection efficiency. Cells were cultured on plates for real-time RT-qPCR and immunoblotting, on cover slips for measurement of intracellular Na^+^ concentration ([Na^+^]_i_) and epifluorescence microscopy, or on filter units for equivalent I_sc_ measurement.

### [Na^+^]_i_ measurement

Single-cell fluorescence imaging using Na^+^ indicator SBFI-AM was conducted to determine the intracellular sodium ion concentration ([Na^+^]_i_), as described by others [37], [38], [39] and us [23]. Briefly, cells transiently transfected with RFP or RFP-AF17 fusion constructs were first identified and selected under epifluorescence microscopy with a filter combination specific for RFP. These cells were then used for collecting SBFI image data under a separate filter combination. In all cases, data from multiple cells in each experiment were represented by the average and counted as a single observation (n = 1). The bath temperature was maintained at 37°C throughout the experiments.

To calibrate the intracellular SBFI-AM dye fluorescence, ionophores (5 µM nigericin + 5 µM monensin) were used to permeabilize the cell membrane and equilibrate [Na^+^]_i_ with bath [Na^+^] ranging from 0 to 140 mM [23], [40]. Calibration curves were generated using non-linear least small squares regression [41].

### I_sc_ measurement

Cells grown on 12-mm filter units were fed on both apical and basolateral sides with culture medium (DMEM/F12 plus 10% FBS) and allowed to form monolayers. The medium was replaced every 3 days. An Epithelial Volt-ohmmeter (EVOM) (World Precision Instruments) with a set of Ag:AgCl electrodes was used to regularly monitor the transepithelial voltage (V_TE_) and transepithelial resistance (R_TE_) of each filter under sterile conditions as reported by others (17, 19) and us [23]. Monolayers with the resistance ≥900 Ω·cm^2^ were considered confluent (17) and used to measure V_TE_ every 1 min for minimal 10 times. Benzamil (1 µM) was then added to the apical side. Five minutes later, V_TE_ was determined again similarly. The readings before or after benzamil administration from a single filter were averaged and counted as 1 (n = 1). The benzamil-sensitive equivalent I_sc_ was determined as the current difference with and without benzamil in the apical bathing solution.

### Epifluorescence and deconvolution microscopy

Cells grown on coverslips were transfected with the plasmids as indicated in figure legends, using Lipofectamine 2000 (Invitrogen). 24 h later, cells were rinsed in phosphate buffered saline, fixed with 1% fresh prepared paraformaldehyde for 30 minute, and stained with 300 nM 4″,6-diamidino-2-phenylindole (DAPI, Sigma) for 15 min. All of these steps were done at room temperature. Coverslips were mounted onto microscope slides with Vectashield mounting medium (Vector Laboratories), and examined either by epifluorescence or deconvolution microscopy. Cells expressing GFP-Dot1a and RFP-AF17 (Fig. 1), or GFP-Dot1a alone (Fig. 2) were categorized as cytoplasmic [C], nuclear [N], or both [C/N], depending on the primary location of the fusion proteins detected by epifluorescence microscopy [18], [42]. It should be pointed out that cells considered as C do not necessarily indicate that the fusion proteins are exclusively located in the cytoplasm. Nevertheless, it does mean that the vast majority of the fusion proteins are located in the cytoplasm. This rule is also applied to N. We then randomly selected multiple fields of each transfection and took images with a deconvolution microscope to confirm the distribution pattern. Deconvolution microscopy was performed at the Multi-User Fluorescence Imaging and Microscopy Core Facility, Department of Pathology and Laboratory Medicine, University of Texas Medical School, Houston, TX. The protocols for image analysis were detailed in our earlier publications [16], [43]. Both epifluorescence microscopy and deconvolution microscopy yielded consistent results in most cases. This may explain why many other groups have used epifluorescence microscopy for similar experiments [44], [45], [46].

### ChIP, real-time qPCR, or RT-qPCR

ChIP assays were performed as detailed previously [15] with the sonication setting (3×10 s, duty cycle: 50% and output control: 6) using a Sonifier Cell Disruptor 350 (Branson). Gradient annealing temperatures were employed to optimize the PCR conditions for each primer pairs, generating a single sharp band with correct size revealed by agarose gel. Real-time qPCRs were assembled with SYBR Green Supermix (Bio-Rad) and conducted on a DNA Engine Opticon System 2 (MJ Research). Each sample was analyzed in triplicate. Serially diluted input DNA derived from a mixture of all samples was used as a positive control and assigned an arbitrary copy number to generate a standard curve for each pair of primers at a fixed threshold cycle detection level. The copy number of each test sample was then automatically calculated according to its threshold cycle value, measured from cycle-dependent product amplification curves. The relative binding of each protein at each region was calculated by measuring the apparent immunoprecipitation efficiency (the ratio of the copy number of the ChIP sample to that of the corresponding input) [15], [47]. For each ChIP, the input contained 3% of the start material. For real-time RT-qPCR, elimination of potential genomic contamination was conducted by pretreatment of total RNA samples with DNase I and verified by RT-PCR in which the reverse transcriptase was omitted (data not shown). cDNA was then synthesized with iScript cDNA Synthesis kit (Bio-Rad) and similarly analyzed with the primers specific for the genes indicated in the figure legends in separate wells. The copy number of each transcript was normalized to that of actin and/or GAPDH from the same sample. The sequences of all primers and the detailed PCR conditions are available from the authors upon request. All experiments were replicated a minimum of three times, with each independent observation representing a single *n* in the figure legends. The triplicates in RT-qPCR or qPCR experiments were counted as a single *n* because they were derived from a single sample, and their average was used to represent the corresponding sample.

### Luciferase and immunoblotting analyses

These assays are routinely used and were conducted according to our published protocols [15], [16], [17].

### Statistical analysis

All quantitative data shown are mean±SEM. For all comparisons, unpaired Student t-test was performed as reported by others and us [16], [17], [34], [38]. P<0.05 is considered as significant.

## Supporting Information

Figure S1 **Overexpression of WT AF17 affects the cellular distribution of GFP-Dot1a.** ***A.*** M-1 cells were transiently transfected with RFP-hAF17 along with pcDNA3.1 (Vec) or its derivative encoding Dot1a, examined with epifluorescence microscopy, and categorized as cytoplasmic (*C*), nuclear (*N*), or both (C/N) depending on the location of RFP-hAF17. The graphed value (%) is the number of cells of each localization type divided by the total number of cells examined. At least 200 cotransfected cells were examined from three independent experiments (*n* = 3). Each percentage was compared within the same category within the category. *n* = 3. No significance was detected at *p*<0.05. ***B*** *.* As in ***A*** except plasmids expressing WT hAF17 and GFP-Dot1a were used for transfection. *: p<0.05.
(EPS)

Figure S2 **Aldosterone does not affect AF17 mRNA expression in M-1 and mIMCD-3 cells.** ***A-B.*** Total RNA was isolated from M-1 cells cultured in DMEM plus 10% charcoal-stripped FBS for at least 50 hrs, then treated with ethanol as vehicle control (-Aldo) or 1 µM aldosterone (+Aldo) for 24 h and analyzed by real-time RT-qPCR for expression of ENaC subunits as positive controls for aldosterone upregulated genes (***A***), and ENaC transcriptional regulators Dot1a, AF9 (as positive controls of aldosterone downregulated genes), and AF17 (***B***). The mRNA level of each gene was first normalized against β-actin mRNA in the same sample, and set to 1 in the vehicle-treated cells. In all cases, n = 3. *: P<0.05 vs. “-Aldo”. ***C.*** As in ***B*** except mIMCD-3 cells were used. n = 3. *: P<0.05 vs. “-Aldo”.
(EPS)

Figure S3 **AF17 knockdown had marginal effects on the aldosterone-mediated induction of** ***β and γENaC*** **expression.** Stably transfected M-1 cells depleting AF17 (see Fig. 2A) were treated with ethanol as vehicle control (-Aldo) or aldosterone (+Aldo, 1 µM), and analyzed by RT-qPCR for β and γENaC as in Fig. 4F. In all cases, n = 3. *: P<0.05 vs. vector.
(EPS)

Figure S4 **Stably transfected M-1 cells developed monolayers with high resistance.** ***A.*** M-1 cells stably transfected with pCDNA3.1 (Vec) or pcDNA-hAF17 (AF17) were grown on permeable filters and allowed to form confluent monolayers. Shown are the resistances before the *I_sc_* measurement as shown in Fig. 8. *n* = 12 or 15 for each cell population, respectively. ***B.*** As in ***A*** except that M-1 cells stably transfected with pSilencer-2.1-U6-Hygro vector (Vec) or its derivative bearing AF17-specific siRNA#11. n = 14 and 17, respectively.
(EPS)
